# Distribution of inhaled volatile β-caryophyllene and dynamic changes of liver metabolites in mice

**DOI:** 10.1038/s41598-021-81181-z

**Published:** 2021-01-18

**Authors:** Yuki Takemoto, Chihiro Kishi, Yuki Sugiura, Yuri Yoshioka, Shinichi Matsumura, Tatsuya Moriyama, Nobuhiro Zaima

**Affiliations:** 1 Department of Applied Biological Chemistry, Graduate School of Agriculture, Kindai University, 204-3327 Nakamachi, Nara, Nara 631-8505 Japan; 2 Department of Biochemistry, Keio University School of Medicine, Tokyo, Japan; 3 Inabata Koryo, Co., Ltd., 3-5-20 Tagawa, Yodogawa, Osaka 532-0027 Japan; 4 Agricultural Technology and Innovation Research Institute, Kindai University, Nara, 631-8505 Japan

**Keywords:** Oils, Gas chromatography, Mass spectrometry

## Abstract

β-caryophyllene (BCP), an essential oil component of many herbs and spices, has various biological activities as a functional food factor. A distinct feature of BCP is its volatile double-ring sesquiterpene structure. Orally administered BCP is reportedly detected in its intact form in mice serum; however, the distribution of inhaled volatile BCP throughout the body remains unknown. This study aimed to estimate the distribution properties of inhaled volatile BCP and to investigate its effects on metabolism. After mice were exposed to volatile BCP, it was detected in the lung, olfactory bulb, brain, serum, heart, liver, kidney, epididymal fat, and brown adipose tissue. BCP was further detected in the brain, liver, and brown adipose tissue 24 h after exposure. Metabolites related to glutathione metabolism were significantly altered in the liver. These results suggest that inhaled volatile BCP is widely distributed in murine tissues and affects the dynamics of metabolites in the liver.

## Introduction

Essential oils have been used to regulate autonomic nervous activity, control depression, anxiety, cognitive disorders, insomnia, and stress-related disorders^1–5^. Several studies conducted at various stages in vitro to in vivo have suggested the pharmacological effects of inhaled components in essential oils^6^. β-caryophyllene (BCP), a volatile bicyclic sesquiterpenoid, is one of the most widely used components in essential oils from hops^7^, black pepper^8^, oregano^9^, basil^10^, rosemary^11^, cinnamon^12^, and cloves^13^. BCP has several bioactivities including anti-inflammatory^14,15^, antioxidant^14,16^, anti-carcinogenic^17,18^, antibacterial^17^, analgesic^18^, local anesthetic^19^, and lipid-lowering activities^20,21^. Gertsch et al. reported that BCP is a selective agonist of the cannabinoid receptor CB2, but not of CB1, and that it can be used for treating various diseases^22^. BCP, is approved as a food flavoring by the US Food and Drug Administration (FDA) and the European Food Safety Authority (EFSA)^18^. Baldissera et al. reported that orally administered BCP can be detected in its intact form in serum^20^. These studies suggest that BCP in food can be distributed in the body and can affect its physiological functions. However, the distribution and effects of inhaled volatile BCP in the body remain unknown.

In this study, we estimated the distribution of BCP and the metabolite dynamics in mice exposed to BCP inhalation. We present analytical evidence indicating that inhaled volatile BCP is widely distributed in mice, and that it affects the dynamics of metabolites in the liver.

## Results

### Serum and organ BCP concentrations in mice

**Figure 1:**
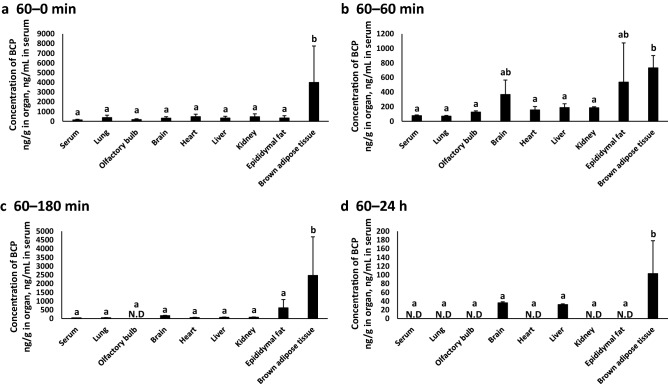
BCP concentrations in mice. **(a)** 60–0 min group (n = 6), **(b)** 60–60 min group (n = 6), **(c)** 60–180 min group (n = 6), **(d)** 60 min-24 h group (n = 6). Values with different letters are significantly different (p < 0.05).

First, we quantified the distribution of BCP in exposed mice under four different conditions: 60–0 min, 60–60 min, 60–180 min, and 60 min–24 h groups (Fig. 1; the experimental scheme is depicted in Supplementary Figure S1a). The concentration of volatile BCP in the flask without mice (just before the start of the exposure experiment) was 2.29 ± 0.17 ng/100 mL of air in the flask. BCP was detected in all organ samples (serum, lung, olfactory bulb, brain, heart, liver, kidney, epididymal fat, and brown adipose tissue). The BCP concentration in brown adipose tissue was significantly higher, or had a higher trend, compared with other samples from all experimental groups. BCP in the olfactory bulb disappeared in the 60–180 min group (Fig. 1c). BCP in the serum, lung, heart, kidney, and epididymal fat disappeared in the 60 min–24 h group, whereas it was consistently detected in the brain, liver, and brown adipose tissue (Fig. 1d). The half-life of BCP concentration in serum was 134.4 min.

### Temporal changes in BCP concentrations in mice

**Figure 2:**
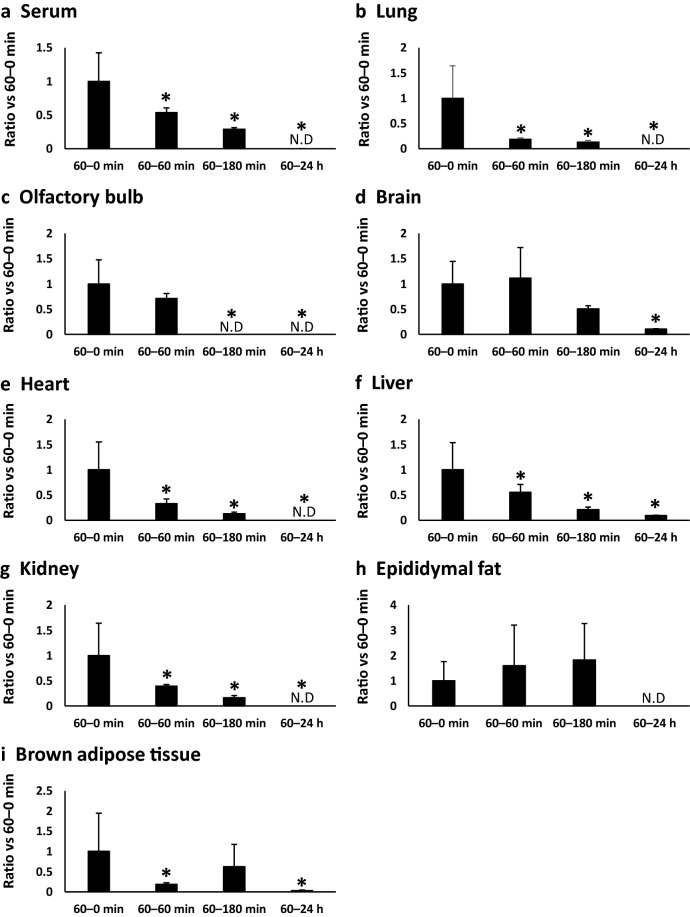
Temporal changes of BCP in mice. **(a)** Serum, **(b)** lung, **(c)** olfactory bulb, **(d)** brain, **(e)** heart, **(f)** liver, **(g)** kidney, **(h)** epididymal fat, **(i)** brown adipose tissue. 60–0 min group (n = 6), 60–60 min group (n = 6), 60–180 min group (n = 6) and 60 min-24 h group (n = 6). Values are present as ratio vs 60–0 min group. Values of BCP in each 60–0 min group were set as 1. Values with different letters are significantly different (p < 0.05).

To estimate the residual properties of BCP in mice, we investigated temporal changes in its levels (Fig. 2). The level of BCP decreased in a time-dependent manner in the serum, lung, heart, liver, kidney, and brown adipose tissue. The levels of BCP in the olfactory bulb remained unchanged until 60 min after exposure (60–60 min group), and decreased significantly in the 60–180 min group (Fig. 2c). The level of BCP in the brain and epididymal fat remained unchanged until 180 min after exposure (60–180 min group), and decreased in the 60 min–24 h group.

### Metabolic variation inferred from metabolome analysis

**Figure 3:**
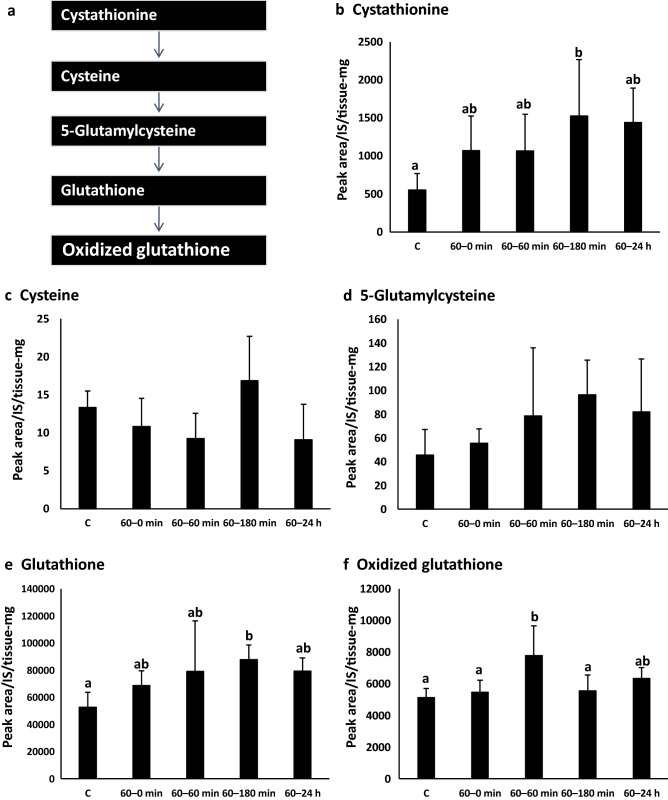
Changes in metabolites associated with glutathione metabolism. **(a)** Glutathione metabolism pathway, **(b)** cystathionine, **(c)** cysteine, **(d)** 5-glutamylcysteine, **(e)** glutathione, **(f)** oxidized glutathione. C group (n = 7), 60–0 min group (n = 6), 60–60 min group (n = 6), 60–180 min group (n = 6) and 60 min-24 h group (n = 6). Values with different letters are significantly different (p < 0.05).

We performed metabolome analysis to evaluate the effects of BCP exposure on metabolism. The results showed an increasing trend involving the glutathione metabolic pathway (Fig. 3). Among the pathway components, cystathionine and glutathione levels in the 60–180 min group were significantly higher than those in the control group, and oxidized glutathione abundance in the 60–60 min group was higher than that in the control group.

### Metabolic variation inferred from volcano plots for metabolome analysis

**Figure 4:**
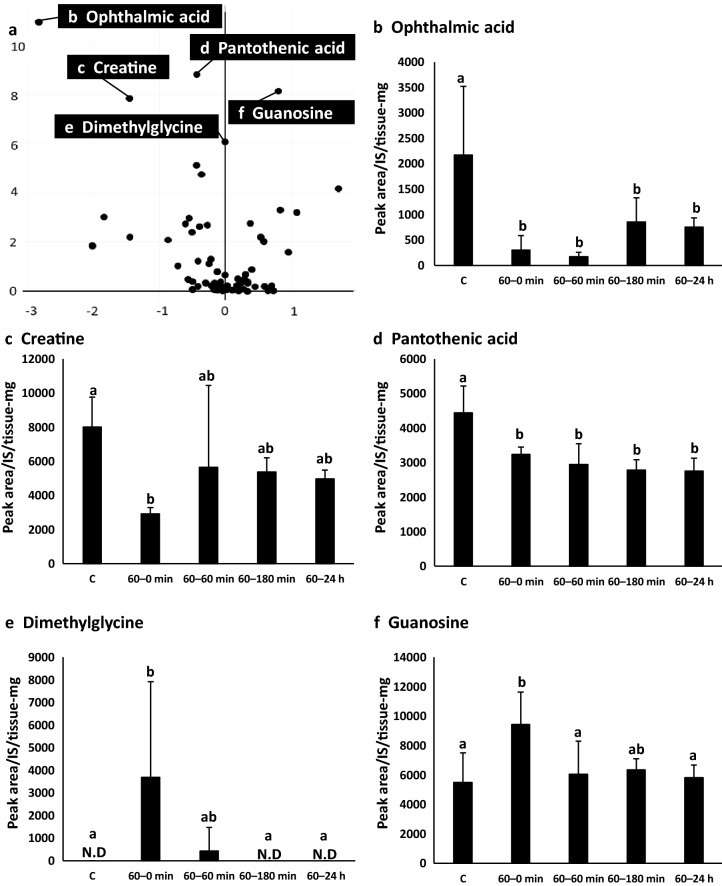
Volcano plot and fluctuating metabolites. **(a)** Volcano plot (compared with C group and 60–0 min group), **(b)** ophthalmic acid, **(c)** creatine, **(d)** pantothenic acid, **(e)** dimethylglycine, **(f)** guanosine. C group (n = 7), 60–0 min group (n = 6), 60–60 min group (n = 6), 60–180 min group (n = 6) and 60 min-24 h group (n = 6). Values with different letters are significantly different (p < 0.05).

Volcano plots revealed changes in metabolites other than those involved in the glutathione metabolic pathway (Fig. 4a). Ophthalmic acid levels in all experimental groups were significantly lower than those in the control group (Fig. 4b). Creatine levels in the 60–0 min group were significantly lower than those in the control group (Fig. 4c). Pantothenic acid levels in all groups were significantly lower than those in the control group (Fig. 4d). Dimethylglycine levels in the 60–0 min group were significantly higher than those in the control group (Fig. 4e). Guanosine levels in the 60–0 min group were significantly higher than those in the control group (Fig. 4f).

## Discussion

Recent studies have reported the biological activities of BCP as a functional food factor^23^. In this study, we focused on volatile BCP and investigated its tissue and organ distribution in exposed mice. Under our experimental conditions, intact BCP was detected in all samples obtained from mice. In addition, metabolome analysis revealed changes in several metabolites in the liver of mice.

It is speculated that volatile BCP is incorporated into the organs of mice via microvessels in the lungs. Detection of BCP in the brain is of particular interest: in general, only a limited number of functional food factors can be distributed to the brain. This is because the blood–brain barrier limits the transfer of substances from the blood to the brain^24^. Our results indicate the possibility that inhaled volatile BCP might be distributed into the brain via blood circulation. Further, Kumar et al. reported the possibility of the distribution of nasally administered pharmaceuticals to both cerebrospinal fluid and blood^25^. Therefore, in addition to the lung pathway, direct distribution via the nasal cavity must also be considered as a potential distribution pathway to the brain. The respiration rate did not change between before inhalation (0 min) and during the inhalation (1 min, 30 min) of volatile BCP (Supplementary Figure S3). BCP levels were higher in brown adipose tissue than in other organs. This may be due to the lipophilic nature of BCP (log p = 4.52). The reason for the high amount of BCP in brown adipose tissue compared to that in white adipose tissue may be the presence of more capillaries in brown adipose tissue than in white adipose tissue^26^. It is thus speculated that a greater quantity of BCP can be taken up from the blood by brown adipose tissue.

Metabolomic analysis of the liver revealed characteristic changes involving metabolites of the glutathione metabolic pathway. Glutathione is a tripeptide consisting of three amino acids (glutamic acid, cysteine, and glycine), and an endogenous antioxidant that donates electrons to free radicals and returns them to stable-state molecules^27^. In this experiment, the level of glutathione in the 60–180 min group was significantly higher than that in the control group. It has been reported that BCP exerts antioxidant effects^14,16^. Distributed volatile BCP might increase the level of glutathione and thereby increase hepatic antioxidant capacity.

Volcano plots revealed changes in metabolites other than the glutathione metabolic pathway. Under oxidative stress, ophthalmic acid is synthesized from 2-aminobutyric acid in a continuous reaction with 5-glutamylcysteine and glutathione synthetase^28^. Ophthalmic acid is excreted from hepatocyte membranes via a multidrug resistance protein transporter^28^. However, under reducing conditions, ophthalmic acid synthesis is suppressed by glutamine feedback^28^. Under oxidative stress, the level of glutathione can decrease, whereas the level of ophthalmic acid can increase. In the experiments reported here, the level of glutathione was increased, and the abundance of ophthalmic acid was decreased after exposure to volatile BCP. In addition, the oxidized glutathione/glutathione ratio was not significantly different across all groups (Supplementary Figure S2). The oxidized glutathione/glutathione ratio has been proposed as a sensitive indicator of oxidative stress^29,30^. These data suggest that volatile BCP is unlikely to cause oxidative stress. The majority (up to 94%) of creatine is found in muscular tissues. When exercising under anoxic conditions, creatine phosphate is rapidly decomposed to transfer phosphate groups to ADP moieties, and ATP is rapidly resynthesized^31^. It is also converted to creatinine through the urea cycle and is metabolized^32^. Pantothenic acid is a vitamin that assists enzymes essential for the metabolism and energy production of sugars, lipids, and proteins^33^. Yoshida et al. reported that administration of large amounts of fat to rats fed a diet containing sufficient vitamins reduced the pantothenic acid content of liver, plasma, and urine^34^. Dimethylglycine is called vitamin B15 or pangamic acid, which is a derivative of the amino acid glycine^35^. Friesen et al. reported that dimethylglycine may be a source of glycine for glutathione synthesis, and may improve the antioxidant capacity of the body^36^. Other researchers have also reported that dietary supplementation with dimethylglycine reduces oxidative stress and improves athletic performance in humans^37^. Guanosine is a nucleoside that exerts neuroprotective effects by antagonizing the neurotoxic and antioxidant properties of glutamate^38^. The changes observed in the above-mentioned metabolites suggest that BCP transferred to the body by inhalation exposure might affect metabolism and physiological functions. However, no drastic morphological changes were observed by upon hematoxylin–eosin staining (Supplementary Figure S4).

In conclusion, this is the first study to reveal the tissue and organ distribution of inhaled volatile BCP in animals. In a time-course analysis, BCP was detected in the brain, liver, and brown adipose tissue up to 24 h after exposure, indicating a long-lasting impact on these organs. The BCP concentrations used for exposure in these experimental conditions were much higher than those to which humans are exposed in daily life. Therefore, the physiological significance of BCP distributed under such conditions should be interpreted independently of the prevailing conditions. However, the results of the experiments presented here indicate the necessity of evaluating the effects on living organisms exposed to environments with high levels of volatile components such as food factories. One limitation of this study was the lack of direct evidence for a relationship between the distributed BCP and the dynamics of liver metabolites. However, these results indicate that volatile BCP can affect metabolites via olfactory receptor-dependent pathways and that further studies are needed to clarify the effects of distributed volatile BCP in living organisms.

## Materials and methods

### Preparation of BCP fractions

To obtain BCP, 150 g of clove leaf oil (Plant Lipids, Kerala, India) and 300 g of potassium hydroxide solution (20% KOH, 80% (v/v) methanol) were combined and reacted at 50 °C for 30 min. After overnight incubation, the separated upper (oil) layer was combined with 100 g of 5% citric acid water and shaken well. The oil layer was separated by centrifugation (8500 × *g*, 5 min) to obtain 3.78 g of the BCP crude fraction. Finally, this was distilled under reduced pressure (110 °C, 150 Pa) to obtain 3.32 g of the BCP fraction. The purity of BCP was 88.5%, as measured by the method described in the GC–MS analysis.

### Animals

All animal experiments were approved by the Institutional Animal Care and Use Committee and were conducted according to the Kindai University Animal Experimentation Regulations (approval number KAAG-31-008). All efforts were made to minimize suffering. Five-week-old male ddY mice (28.2 ± 0.17 g) (Japan SLC, Inc., Shizuoka, Japan) were provided with free access to a commercial diet (MF; Oriental Yeast Co., Ltd., Tokyo, Japan) and water. They were housed at 25 ± 1 °C on a standard 12 h light/dark cycle.

### Experimental conditions for evaluation of BCP distribution in mice

After 5 days of habituation, the mice were divided into 5 groups. A negative control group (no exposure, n = 7), a 60–0 min group (volatile BCP exposure for 60 min and breathing for 0 min in normal air (n = 6)), a 60–60 min group (volatile BCP exposure for 60 min and breathing for 60 min in normal air (n = 6), 60–180 min group (volatile BCP exposure for 60 min and breathing for 180 min in normal air (n = 6)), and a 60–24 h group (volatile BCP exposure for 60 min and breathing for 24 h in normal air (n = 6)). The experimental scheme is shown in Supplementary Figure S1 (a). The mice in each group were placed in a flask for 60 min, with or without volatile BCP (Supplementary Figure S1 (b)). In preparation for the volatile BCP exposed group, BCP was soaked in absorbent cotton and inserted into a net. Next, the absorbent cotton was hung in a flask, and the flask was closed with a silicone stopper for 10 min to fill it with volatile BCP. After 10 min, mice were anesthetized using sodium pentobarbital (50 mg/kg, intraperitoneal injection, Nacalai Tesque, Inc., Kyoto, Japan) and placed in the flask. After 60 min of exposure, the mice were allowed to breathe under normal air.

### Sample collection

Mice were euthanized by an overdose of the anesthetic pentobarbital sodium, and blood samples were obtained via the inferior vena cava. The left cardiac ventricle of mice was subsequently perfused with an isotonic sodium chloride solution. The lung, olfactory bulb, brain (cerebrum and cerebellum), heart, liver, kidney, epididymal fat, and brown adipose tissue samples were isolated and subsequently frozen on a plate cooled with liquid nitrogen, without any prior chemical fixation. These organ samples were stored at − 80 °C until further analysis by gas chromatography-mass spectrometry (GC–MS).

### Experimental conditions for evaluating the BCP concentration in flask

BCP concentration in the flask was measured using a GC–MS (Agilent 7890A-5975C Mass Selective Detector; Agilent Technologies, Santa Clara, CA, USA). BCP (10 mL) was soaked in absorbent cotton and placed in a net (mesh: 0.18 cm^2^, Hatto Co., Wakayama, Japan). It was hung in the flask using a paper string (Kouyu Co., Ltd., Fukushima, Japan), and the flask was closed with a silicone stopper. After 10 min, BCP was absorbed using an InertSep C18 cartridge (200 mg/mL, GL Science Inc., Tokyo, Japan) at 3 cm from the bottom of the flask to sample 100 mL of air. The absorbed BCP in the InertSep C18 cartridge was extracted with 1 mL of methanol. For GC–MS analysis, an InertCap Pure WAX column (length: 60 m, df: 0.25 μm, inner diameter (I.D): 0.25 mm, GL Sciences, Inc., Tokyo, Japan) was used for separation. The oven temperature was programmed as follows: initial temperature, 50 °C; ramp rate: 2.5 °C/min (50 to 145 °C) and 10 °C/min (124 to 240 °C); and final temperature, 240 °C for 5 min. The He inlet pressure was controlled with an electronic pressure control system to achieve a constant column flow of 1.0 mL/min. MS analysis was performed in the electron ionization (EI) mode at a voltage of 70 eV. Using the peak area of caryophyllene, the caryophyllene concentration/100 mL was determined from the standard curve. The quantity of caryophyllene was determined from the total ions (scan: *m/z* 25–*m/z* 350).

### Experimental conditions for evaluation of BCP concentration in serum and organ samples

BCP concentrations in serum and organ samples were analyzed using a GC–MS (Agilent 7890B-5977B MSD; Agilent Technologies, Santa Clara, CA, USA), a Thermal Desorption Unit (TDU2), Programmable Temperature Vaporization inlet (CIS4), and Multipurpose Sampler (MPS) with Dynamic Head Space (DHS) option (Gerstel GmbH & Co.KG, Mülheim an der Ruhr, Germany)^39^. An MSD ChemStation vs. F. 01.03. 2357 (Agilent) and Mass Hunter v.B.07.05.2479 (Agilent) were used for data analysis.

Tenax TA cartridges were released from Tenax TA traps using a thermal desorption cold-trap setup (thermal desorption spectrometer (TDS); Markes International, Ltd., Llantrisant, RCT, UK). An InertCap WAX column (length: 60 m, df: 0.25 μm, I.D.: 0.25 mm, GL Sciences, Inc., Tokyo, Japan) was used for component separation. The oven temperature was programmed as follows: initial temperature, 40 °C; ramp rate, 3 °C/min (40 to 145 °C) and 10 °C/min (145 to 240 °C); and final temperature, 240 °C for 5 min. The He inlet pressure was controlled using an electronic pressure control system to achieve a constant column flow of 1 mL/min. MS analysis was performed in the ionization mode at a voltage of 70 eV.

### Metabolome analysis

All samples were analyzed by ultra-performance liquid chromatography-mass spectrometry (UPLC–MS) system (LCMS-8050, Shimadzu, Kyoto, Japan). For metabolome analysis, the experimental conditions were as follows: column: Discovery HS F5-3 (length, 150 mm; I.D., 2.1 mm; particle size, 3 μm; Sigma-Aldrich P/N, Tokyo, Japan); flow rate, 0.25 mL/min; mobile phase A, 0.1% formic acid/water; mobile phase B, 0.1% formic acid/acetonitrile; oven temperature, 40 °C; and injection volume, 3 μL. The electrospray ionization mass spectrometry (ESI–MS) parameters were as follows: interface temperature, 300 °C; desolvation line (DL) temperature, 250 °C; heat block temperature, 400 °C; nebulizer gas flow rate, 3 L/min; heating gas flow rate, 10 L/min; and drying gas flow rate, 10 L/min. The multiple reaction monitoring (MRM) mode was used to monitor the transition of Cystine (*m/z*: 241.00 > 151.95), Cystathionine (*m/z*: 223.00 > 88.05), Dimethylglycine (*m/z*: 104.10 > 58.05), Creatine (*m/z*: 132.10 > 44.05), 5-Glutamylcysteine (*m/z*: 251.10 > 84.10), Glutathione (*m/z*: 308.00 > 179.10), Ophthalmic acid (*m/z*: 290.10 > 58.10), Guanosine (*m/z*: 284.00 > 152.00), Pantothenic acid (*m/z*: 220.10 > 90.15), and Oxidized glutathione (*m/z*: 611.10 > 306.00). For each metabolite, the ratio between the test groups was log2 converted to the horizontal axis and the p-values of the t-test results were converted to log10; a volcano plot, which is the scatter plot shown on the vertical axis, was used to compare significant differences between groups. A statistically significant high- or low-metabolite group was estimated.

### Pharmacokinetic analysis in serum

The elimination half-life (t_1/2_) was calculated from the relationship t_1/2_ = 0.693/kel.

### Statistical analyses

Values are expressed as mean ± standard deviation (SD). Statistical differences were determined using the Scheffe test or Dunnett test. P-values < 0.05 were considered statistically significant. Statistical analyses were performed using Stat View v.5.0 software (SAS Institute, Cary, NC, USA).

## Supplementary Information

Supplementary Figures.

Supplementary Legends.
