# Supplementary material for: Distribution of inhaled volatile β-caryophyllene and dynamic changes of liver metabolites in mice
Source: Sci Rep. 2021 Jan 18;11:1728. doi: 10.1038/s41598-021-81181-z (PMC7813867; doi:10.1038/s41598-021-81181-z)

Title: Distribution of inhaled volatile β-caryophyllene and dynamic changes of liver metabolites in mice

Short title: Distribution of inhaled β-caryophyllene and changes of liver metabolites in mice

Yuki Takemoto[1], Chihiro Kishi[1], Yuki Sugiura[4], Yuri Yoshioka[2], Shinichi Matsumura[2], Tatsuya Moriyama[1, 3], Nobuhiro Zaima[1, 3*]

[1] Department of Applied Biological Chemistry, Kindai University, 204-3327 Nakamachi, Nara City, Nara 631-8505, Japan

[2] INABATA KORYO, Co., Ltd., 3-5-20 Tagawa, Yodogawa, Osaka 532-0027, Japan

[3] Agricultural Technology and Innovation Research Institute, Kindai University, Nara, 631-8505, Japan

[4] Department of Biochemistry, Keio University School of Medicine, Tokyo, Japan.

*Corresponding author

Nobuhiro Zaima zaima@nara.kindai.ac.jp

Department of Applied Biological Chemistry, Graduate School of Agriculture, Kindai University, 204-3327 Nakamachi, Nara City, Nara 631-8505, Japan

Keywords

β-caryophyllene (BCP); bioavailability; metabolome; glutathione; volatile component

Supplementary Figure S1

a Experimental scheme

4 weeks ♂ ddY

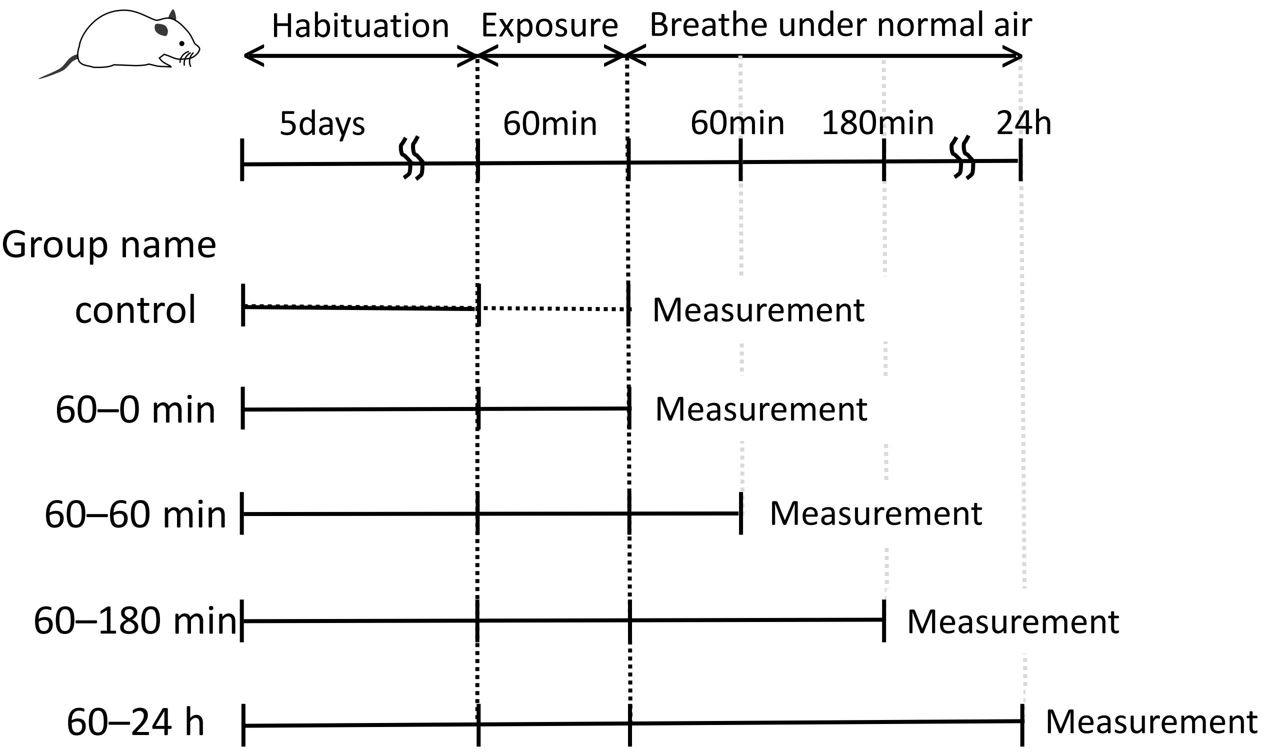

b Method for exposure and breathe under normal air

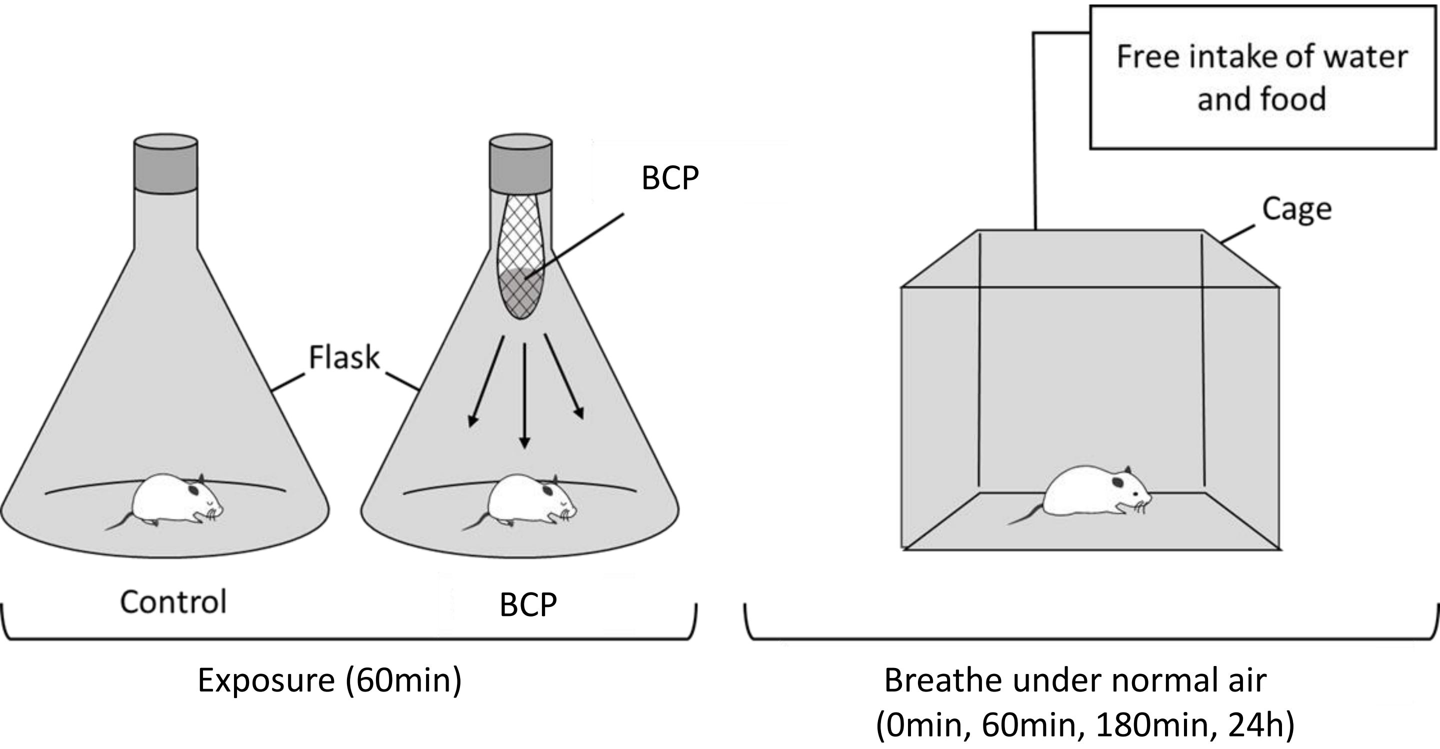

Supplementary Figure S2

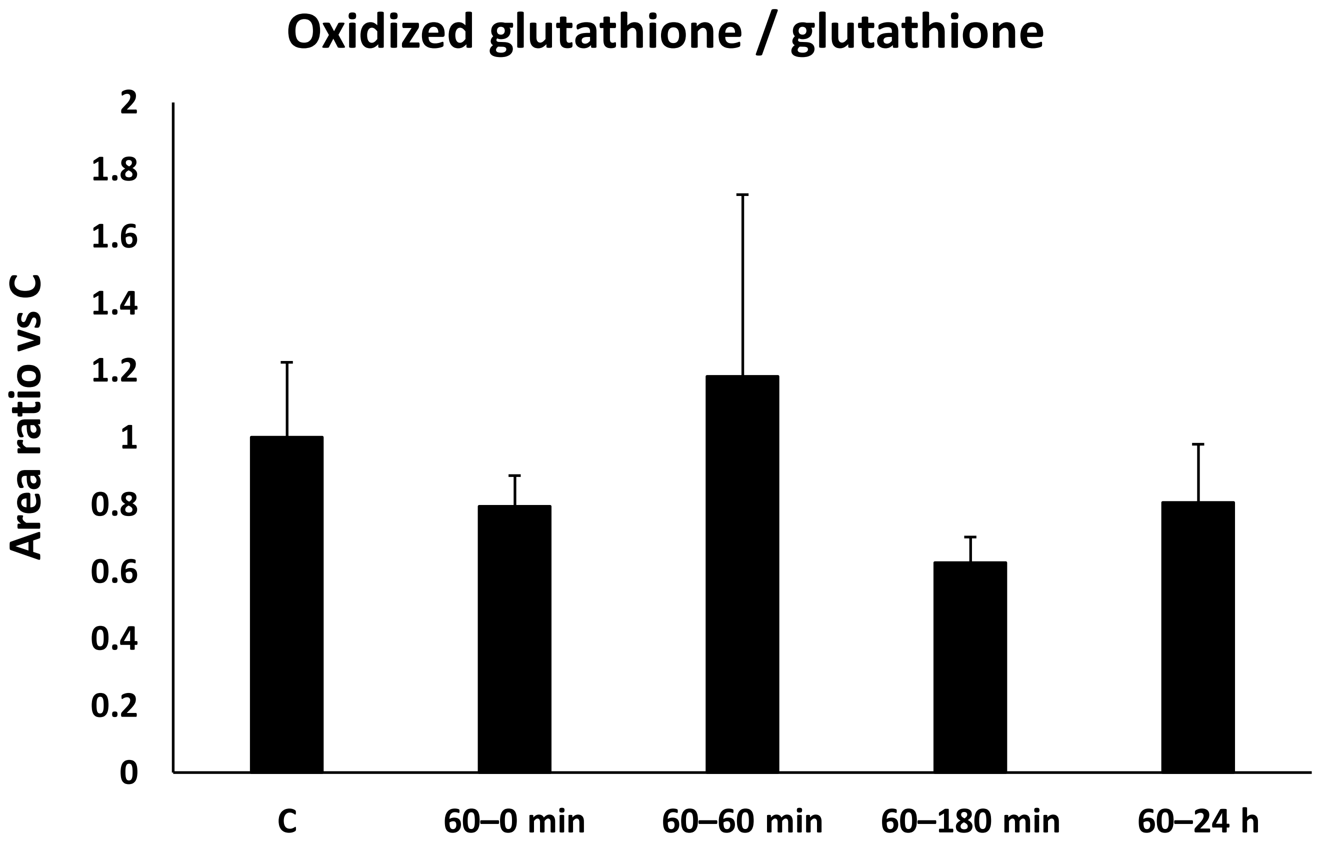

Supplementary Figure S3

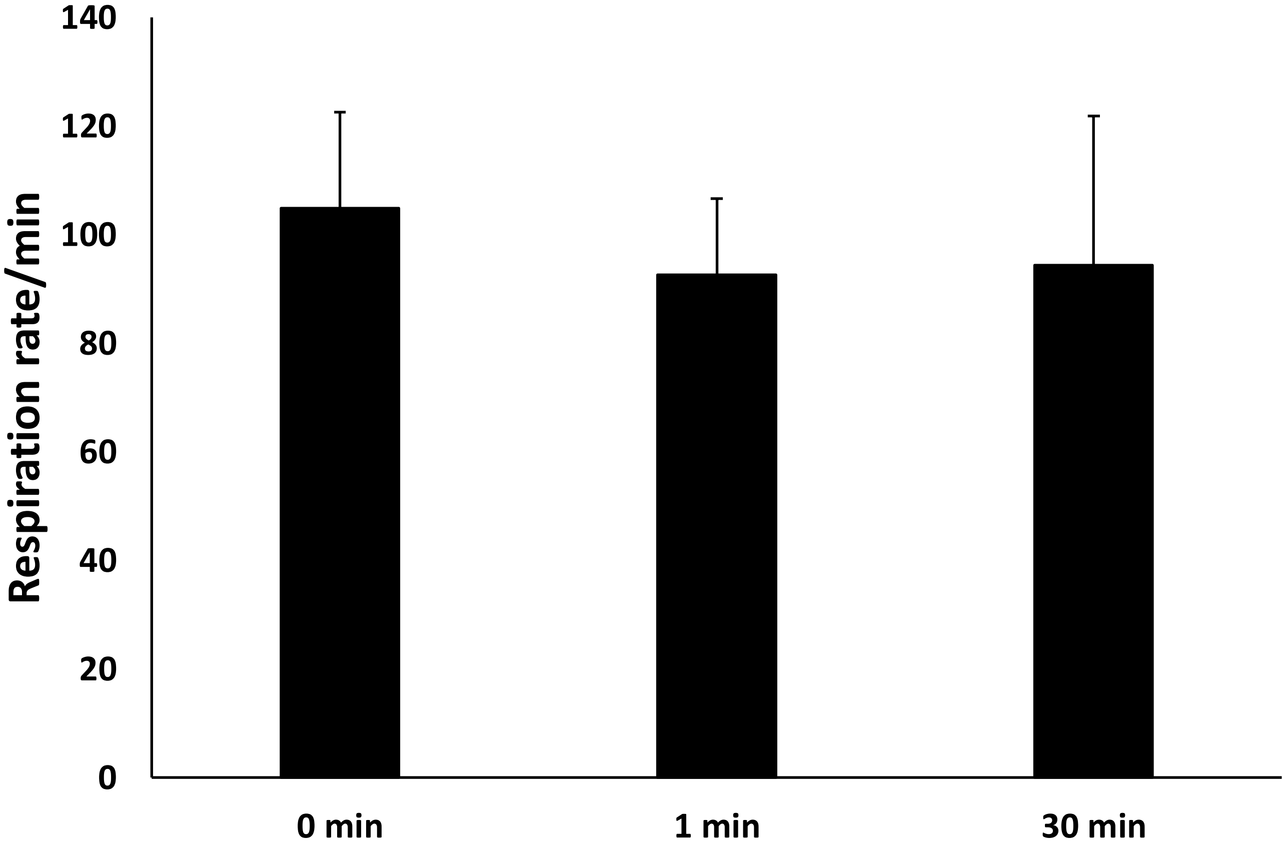

Supplementary Figure S4

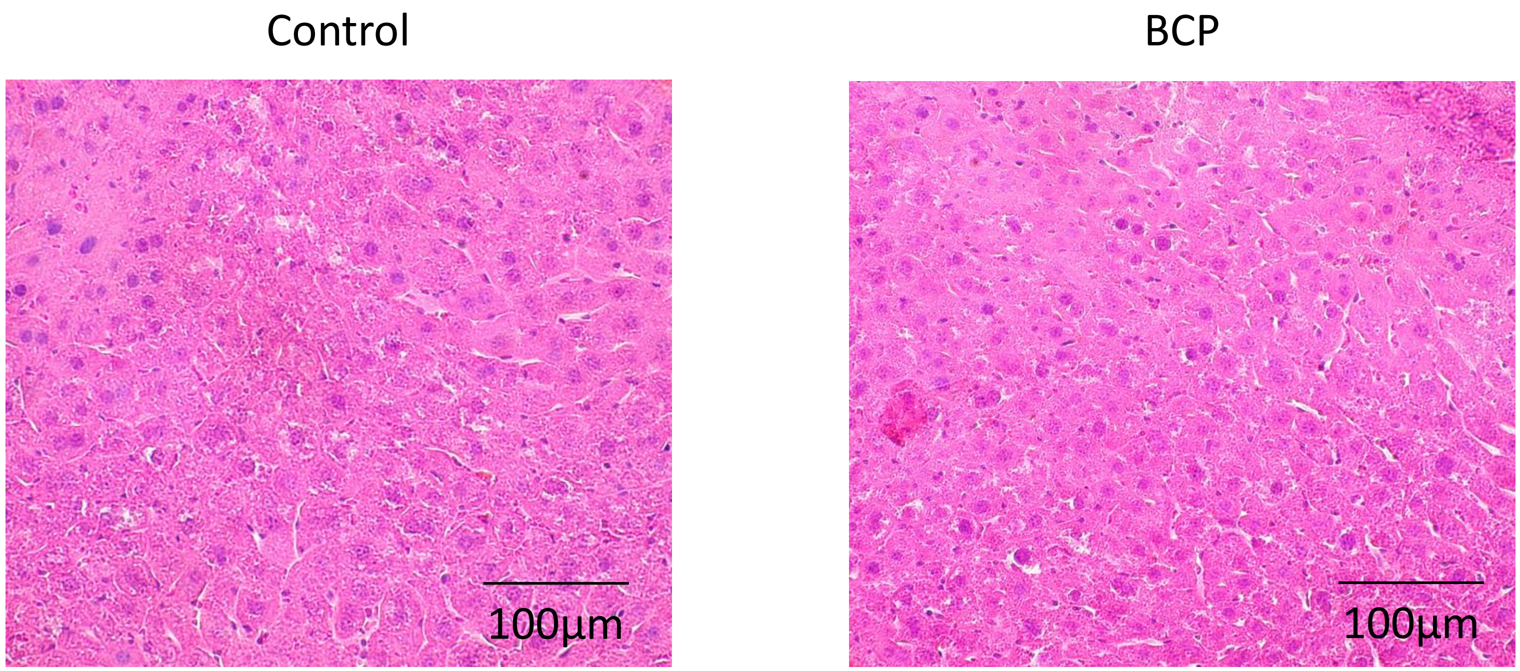

Supplement: Supplementary file 1 — Supplementary Figures. [file 41598_2021_81181_MOESM1_ESM.pdf]
