# Supplementary material for: Distribution of inhaled volatile β-caryophyllene and dynamic changes of liver metabolites in mice
Source: Sci Rep. 2021 Jan 18;11:1728. doi: 10.1038/s41598-021-81181-z (PMC7813867; doi:10.1038/s41598-021-81181-z)
Supplement: Supplementary file 2 — Supplementary Legends. [file 41598_2021_81181_MOESM2_ESM.docx]

**Supplementary Figure S1** Experimental procedures.

1. Experimental scheme of this study (b) Method for exposure to volatile BCP and breathe under normal air.

**Supplementary Figure S2** Sensitive indicator of oxidative stress.

C group (n = 7), 60-0 min group (n = 6), 60-60 min group (n = 6), 60-180 min group (n = 6) and 60 min-24 h group (n = 6). Values are present as Area ratio vs C group. Values of area ratio of oxidized glutathione / glutathione in each C group were set as 1. Values with different letters are significantly different (p < 0.05).

**Supplementary Figure S3** Respiration rate.

0 min (n = 9), 1 min (n = 9) and 30 min (n = 9). Respiration rate per 1 min. Values with different letters are significantly different (p < 0.05).

**Supplementary Figure S4** Hematoxylin-Eosin (HE) staining of liver.

C group (n = 7) and BCP group (n = 8).
